# System-Level Integration and Evaluation of an APS-SoC-Based Electrical Resistance Tomography Measurement System

**DOI:** 10.3390/s26154951

**Published:** 2026-08-05

**Authors:** Donghua Luo, Zhaoyou Han, Shiyuan Zhu, Shihong Yue

**Affiliations:** 1 Shandong Communication & Media College, Jinan 250200, China; luodonghua@sdcmc.edu.cn; 2 Shandong Port Group Co., Ltd., Qingdao 266000, China; hzluobo@126.com; 3 School of Electrical and Information Engineering, Tianjin University, Tianjin 300072, China; shiyuanzhu@tju.edu.cn

**Keywords:** electrical resistance tomography, APS-SoC, ZYNQ-7020, PL/PS co-design, Gigabit Ethernet, Butterworth filter

## Abstract

This study presents and evaluates a system-level optimization of an electrical resistance tomography (ERT) measurement platform based on a ZYNQ-7020 all-programmable system-on-chip (APS-SoC). The design combines deterministic programmable-logic (PL) acquisition, processing-system (PS) configuration and communication scheduling, AXI/DMA data movement, Gigabit Ethernet transmission, and a seventh-order Butterworth excitation filter. FFT-based amplitude extraction and Tikhonov reconstruction remain on the host computer so that the reconstruction algorithm and regularization settings remain identical for the baseline and proposed systems; the present prototype is therefore not claimed as a fully standalone smart sensor. Under the same 16-electrode tap-water testing configuration, the average frame rate increased from 58.23 ± 1.88 FPS to 123.02 ± 1.62 FPS (mean ± sample standard deviation, n = 10), end-to-end latency decreased from 5.2 ms to 2.1 ms, SFDR increased from 68 dB to 95 dB, and SSIM increased from 0.72 to 0.94. In one representative static hardware record at 160 kHz, the calculated amplitude-stability SNR values were 65 dB and 100 dB for the baseline and proposed excitation paths, respectively, while total harmonic distortion decreased from 15.2% to 7.5%. These single-condition signal quality values are descriptive rather than uncertainty-bounded performance specifications. The image-quality differences are attributed primarily to cleaner boundary-voltage measurements with an unchanged reconstruction method, whereas the frame-rate gain reflects the combined PL/PS data path and Gigabit Ethernet upgrade.

## 1. Introduction

Electrical resistance tomography (ERT) [1,2] is a non-invasive, radiation-free, and relatively low-cost technique for estimating conductivity distributions from boundary measurements. A typical ERT instrument comprises an electrode array, a data-acquisition and control unit, and a host computer [3]. The acquisition and control unit integrate excitation generation, electrode selection, signal conditioning, sampling, timing control, and communication; consequently, its architecture and implementation strongly affect measurement quality and usable frame rate [4,5].

Recent FPGA- and system-on-chip-based ERT/EIT systems have improved excitation flexibility, parallel acquisition, and real-time imaging performance [6,7,8,9,10,11,12]. However, many existing studies [13,14,15,16] emphasize one subsystem, such as waveform generation, demodulation, or reconstruction acceleration, whereas fewer studies have reported how excitation quality, deterministic acquisition, on-chip data movement, external communication, and reconstruction-oriented data flow can be jointly optimized within a compact ERT platform. The research question of this work is therefore whether an APS-SoC-based hardware/software co-design [17,18,19,20,21,22] can simultaneously improve signal quality and frame rate in a 16-electrode ERT sensor while preserving a compact hardware structure.

To position the proposed system relative to recent FPGA- or APS-SoC-based ERT/EIT systems, Table 1 summarizes representative studies published from 2020 to 2024 [23,24,25,26]. Information that was not explicitly reported in the corresponding studies is marked as N/R. Compared with these representative systems, the proposed design emphasizes system-level integration by combining ZYNQ-7020 APS-SoC-based PL/PS cooperation, DMA data movement, Gigabit Ethernet transmission, a seventh-order Butterworth excitation filter, and reconstruction-oriented high-speed acquisition within a compact ERT measurement platform.

Because electrode geometry, excitation strategy, sampling protocol, reconstruction method, and reported metrics differ among the cited studies, Table 1 is used only to contextualize the system architecture; the numerical values are not treated as a controlled head-to-head benchmark.

As operating environments and measurement requirements become more demanding, ERT instruments may be limited by communication throughput, non-deterministic data movement, excitation distortion, and electromagnetic interference [6,7]. Legacy laboratory systems often use serial communication links whose practical throughput and noise immunity depend on the protocol, cable length, grounding, and physical-layer implementation [8]. In addition, equipment producing strong alternating magnetic fields can couple interference into excitation and sampling circuits, causing measurement fluctuations and spurious voltage components [9,10].

The baseline system is the authors’ laboratory FPGA-based ERT prototype, not a proxy for all contemporary ERT systems. For the controlled comparison, both systems used the same 16-electrode sensor, adjacent switching protocol, excitation frequencies, 20 MS/s experimental sampling rate, 2048 samples per boundary measurement, host-side FFT amplitude extraction, Tikhonov reconstruction, and evaluation procedure. The baseline used a discrete FPGA controller, RS-485 transmission configured at 2 Mbps, and second-order Sallen-Key filtering, whereas the proposed system used ZYNQ-7020 PL/PS cooperation, AXI/DMA data movement, Gigabit Ethernet, and a seventh-order Butterworth excitation filter. The comparison therefore quantifies the difference between two complete laboratory prototypes under matched sensing and reconstruction conditions. The two filter topologies are soldered onto their respective fabricated PCBs and cannot be interchanged on a common board without redesign and refabrication. Likewise, the RS-485 and Gigabit Ethernet paths are implemented on different controller boards and firmware stacks. Consequently, the analog, controller, and communication changes are not treated as independently isolated factors.

Relative to the laboratory discrete-FPGA implementation, the APS-SoC architecture has the following integration characteristics:

(1) A heterogeneous APS-SoC-based ERT architecture is implemented, in which the programmable logic (PL) performs timing-critical excitation control and synchronous acquisition, while the processing system (PS) performs system configuration, DMA management, and communication scheduling [11]. FFT-based amplitude extraction and image reconstruction are retained on the host computer for two reasons: it keeps the reconstruction code and regularization settings identical to those of the baseline, thereby isolating measurement-chain effects, and the present prototype did not include ARM-optimized reconstruction code or a measured PS CPU/power profile. Thus, the current implementation is not claimed as a fully standalone smart sensor; the available PL resources and PS/PL data path provide a basis for future embedded demodulation or reconstruction.
(2) An on-chip AXI interconnection is used for PL-to-PS data movement [12]. Compared with a separate FPGA-plus-processor implementation, this integration can reduce inter-chip transfers and board-level complexity. However, PCB area and power consumption were not directly measured against the baseline in this prototype; therefore, no quantitative area or energy reduction is claimed [13].
(3) A hardware–software co-design and reconfiguration strategy is developed. The PL can be dynamically updated online to switch among different excitation or measurement modes, while the PS adjusts algorithmic parameters accordingly. This co-optimization mechanism shortens the development and debugging cycle and enhances the flexibility and scalability of the ERT system [14].

Overall, the proposed design provides a unified and reconfigurable platform for acquisition, control, data movement, and communication. Its contribution is system-level integration and optimization rather than a new ERT reconstruction method or the simple replacement of an FPGA device.

The specific novelty of the proposed design is therefore not the simple replacement of an FPGA device, but the coordinated APS-SoC-based architecture that links PL acquisition, PS data management, Gigabit Ethernet transmission, and high-order excitation filtering within a compact ERT measurement platform.

## 2. System Analysis

**Figure 1:**
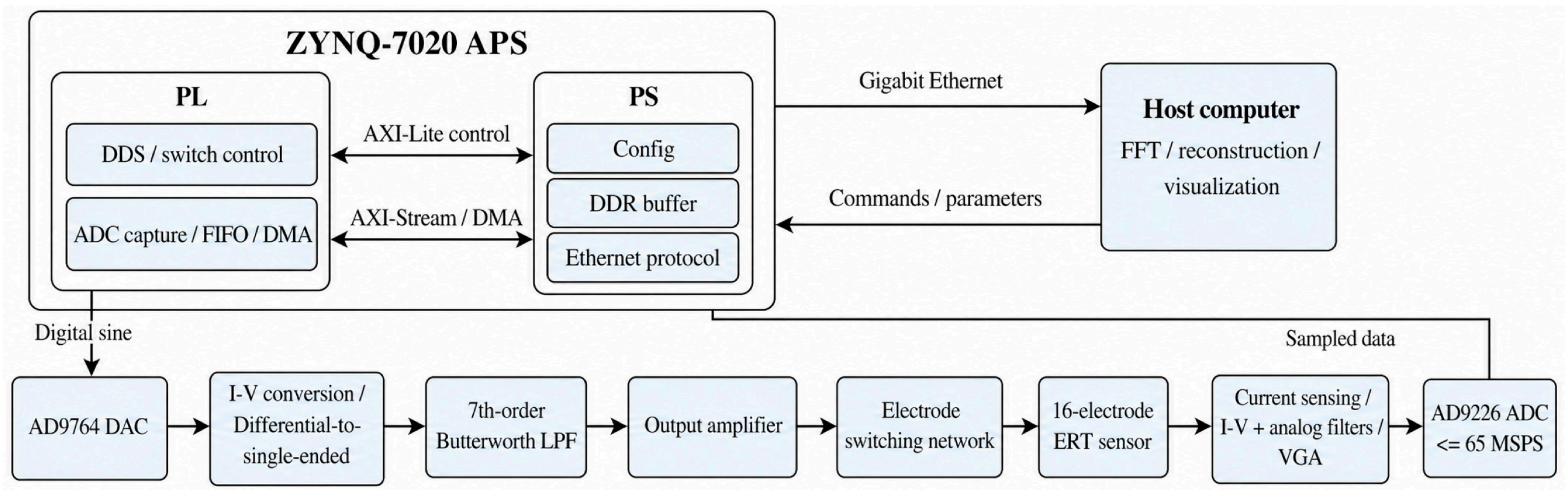
Conceptual architecture of the proposed APS-SoC-based ERT system.

The data-acquisition and control system of ERT typically employs high-speed analog-to-digital converters in the sampling module and programmable logic devices in the main control module. In theory, these components can support rapid measurement and strong real-time capability; in practice, communication bottlenecks, non-deterministic data movement, excitation distortion, and analog-front-end noise often limit the usable frame rate and reconstruction stability. Figure 1 shows the conceptual architecture of the proposed APS-SoC-based ERT system, including PL/PS cooperation, DAC excitation, current-to-voltage conversion, Butterworth filtering, output amplification, electrode switching, analog filtering/amplification, ADC acquisition, DMA transfer, Ethernet communication, and host-computer reconstruction.

However, many legacy systems use serial communication protocols such as RS-232 or RS-485, which can constrain the communication rate between the controller and host computer and create a data-transfer bottleneck. High-power variable-frequency motors, switching equipment, and other sources of electromagnetic interference can also degrade excitation and sampling signals [15,16]. Second-order Sallen-Key filters are commonly used in excitation and analog-front-end circuits, but their gradual roll-off may provide insufficient suppression of high-frequency feedthrough for the present hardware configuration.

To address these engineering bottlenecks, the proposed design integrates control, acquisition, on-chip data movement, and communication in a ZYNQ-7020 APS-SoC [17]. The AXI data path and RTL8211E Gigabit Ethernet interface are used to reduce internal and external data-transfer bottlenecks; the measured frame-rate improvement therefore reflects the combined architecture and communication upgrade, not the ZYNQ device alone.

### 2.1. Main Control Module Design

The ZYNQ-7020 APS-SoC is used as the central controller. It combines a processing system (PS) containing dual ARM Cortex-A9 processors with Artix-7 programmable-logic (PL) resources. The PS and PL exchange data through AXI interfaces. Custom Verilog IP cores were developed in Xilinx Vivado for the PL, and the PS software was developed using the Xilinx Software Development Kit (2019.1). The PS configures the measurement sequence and communication interface, whereas the PL executes timing-critical excitation control, electrode switching, and synchronous acquisition.

**Figure 2:**
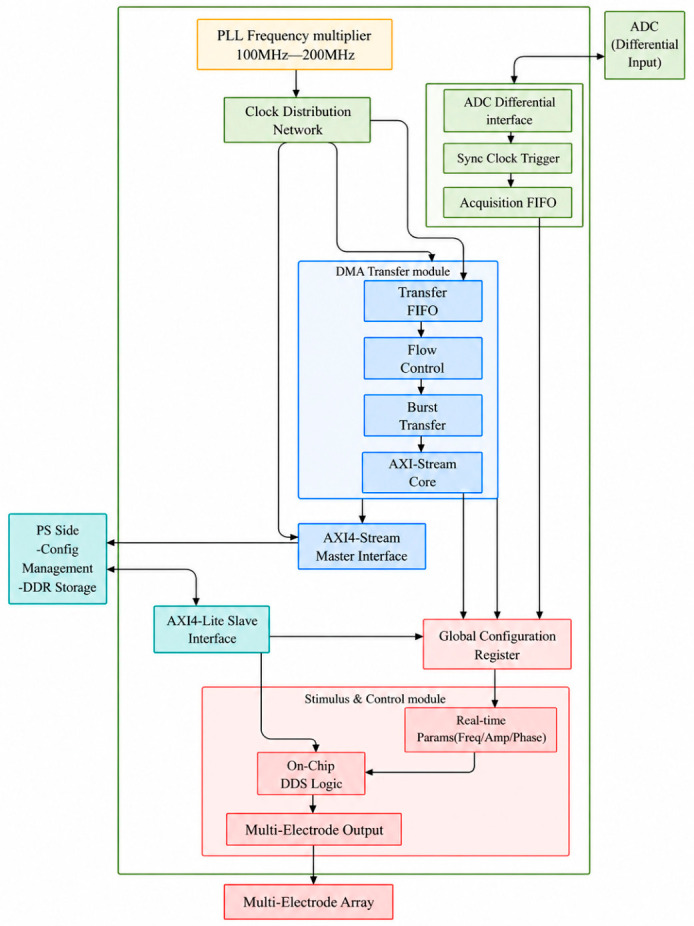
PL diagram.

Within the PL, the design is organized into three core functional modules: the data acquisition module, the excitation/control module, and the DMA transfer module. The PL diagram is shown in Figure 2.

The data-acquisition module captures differential ADC data and performs synchronous sampling with FIFO buffering. The excitation/control module generates parameterizable sinusoidal waveforms and electrode-switching control words; the PS modifies frequency, amplitude, phase, sampling length, and switching sequence through memory-mapped registers. The DMA path uses a 32-bit AXI4-Stream interface clocked at 100 MHz. For the imaging test, each boundary measurement contains 2048 samples that are stored as 16-bit values and packed into burst transfers to PS-accessible DDR memory. A PL-to-PS data-arrival interrupt triggers PS-side buffer handling and Ethernet packet scheduling. Reference [8] reported the corresponding resource and throughput calculations; these are implementation parameters rather than a component-wise latency measurement.

**Figure 3:**
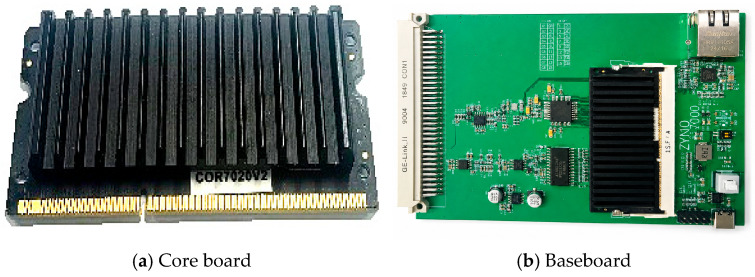
Core board and baseboard of the proposed APS-SoC-based ERT prototype.

All PL control modules operate from a 100 MHz system clock. The PL PLL can generate higher-frequency internal timing clocks for logic synchronization and DAC-related timing; however, the practical ADC sampling rate is determined by the external AD9226 converter, whose maximum rate is 65 MS/s. Therefore, any reference to 200 MHz denotes only an internal PL clock-generation capability, not the AD9226 sampling rate. In the experiments, the AD9226 sampling frequency was configured as 20 MS/s, which is sufficient for the 100–160 kHz excitation signals used in this study and remains below the converter limit. The development board provides DDR3 memory, QSPI, eMMC, and high-density PL expansion connectors. The application baseboard integrates the RTL8211E Gigabit Ethernet PHY, excitation and sampling interfaces, electrode-switching connections, and DC/DC power supplies. Figure 3 shows the core board and baseboard. Any discussion of reduced board-level complexity refers to the integration potential of an APS-SoC relative to a separated FPGA-plus-processor architecture; PCB area was not measured against the baseline.

### 2.2. Excitation Module Design

The excitation module generates the sinusoidal excitation signal and delivers it to the electrode-switching network. Its waveform quality directly affects the boundary-voltage measurements and thus the reconstructed image quality. The excitation path converts the digital sine-wave data generated by the main control module into an analog signal, performs current-to-voltage and differential-to-single-ended conversion, suppresses high-frequency distortion with a seventh-order Butterworth low-pass filter, and then amplifies the signal before electrode injection. Unless otherwise stated, a 100 kHz, 4 Vpp sinusoidal voltage signal is used for the waveform and frame-rate tests.

**Figure 4:**
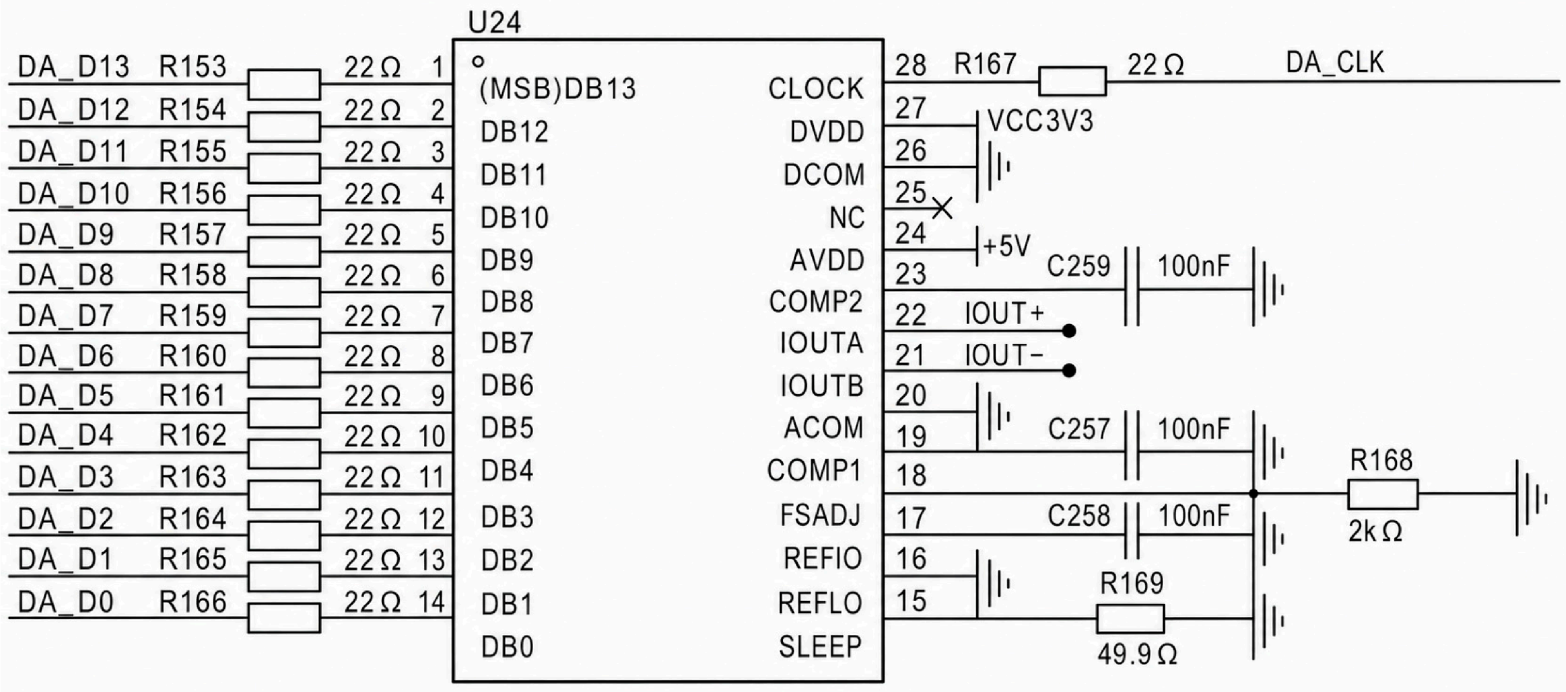
Schematic of the AD9764 D/A converter.

The excitation signal is generated using the AD9764 high-speed digital-to-analog converter from Analog Devices Inc. The device provides 14-bit resolution and a maximum conversion rate of 125 MSPS, which meets the system requirements. The AD9764 provides a differential current output of 2–20 mA, requiring current-to-voltage and differential-to-single-ended conversion. The schematic diagram of the AD9764 and its peripheral circuitry is shown in Figure 4.

**Figure 5:**
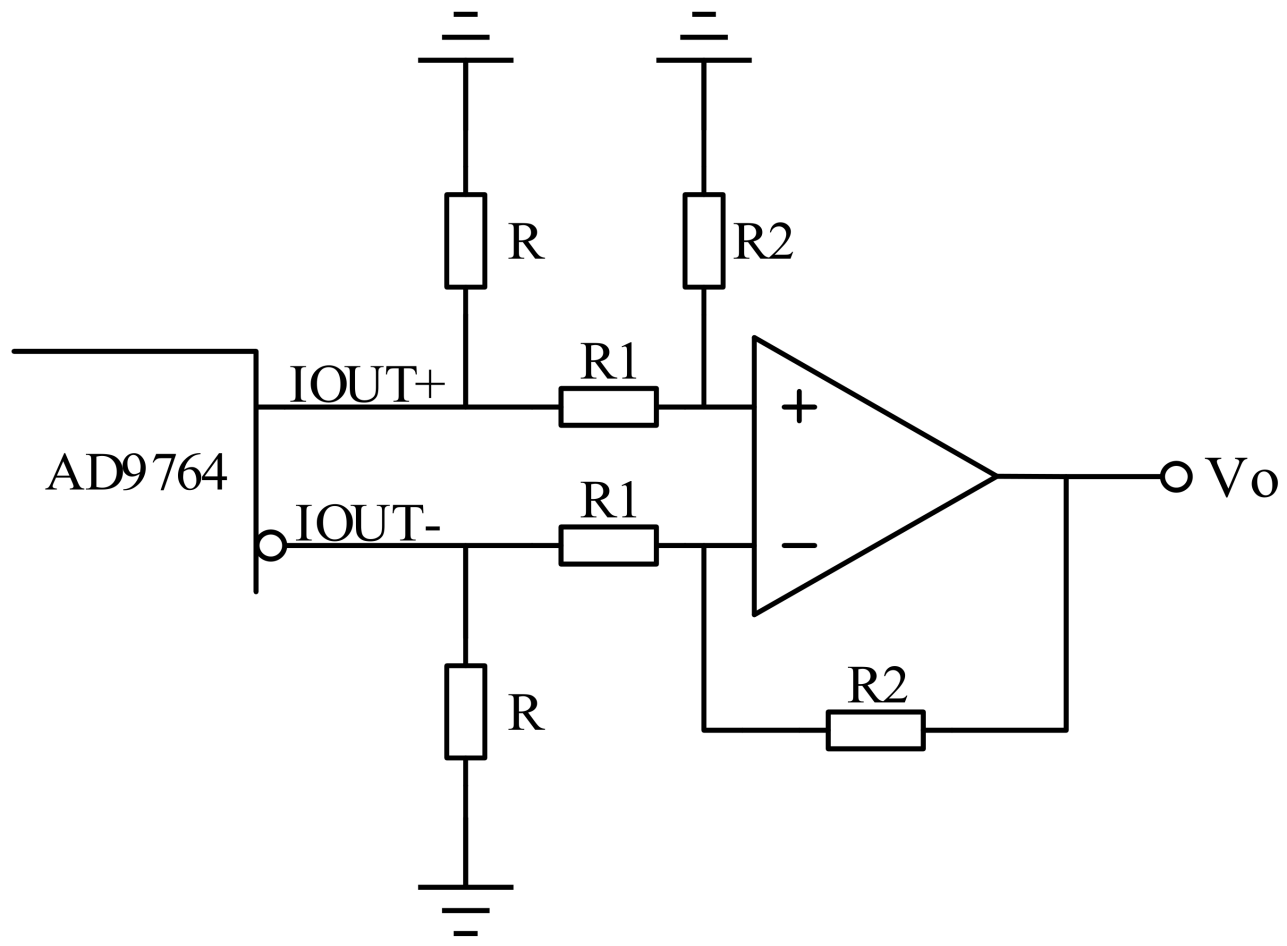
Current-to-voltage and differential-to-single-ended conversion circuit.

In Figure 4, the FSADJ pin is connected to a 2 kΩ resistor, setting the maximum amplitude of the output current to 20 mA. In addition, all AD9764 digital input interfaces are equipped with 22 Ω matching resistors to reduce ringing in the digital input signals. The current-to-voltage conversion and differential-to-single-ended conversion circuits of the AD9764 output are illustrated in Figure 5.

By connecting two resistors in parallel, denoted *R*, at the differential output terminal, the differential current signal is transformed into a differential voltage signal, as shown in Equation (1). Using a differential amplifier constructed with the high-speed operational amplifier OPA690, the differential signal is converted to a single-ended signal, with the output voltage at this stage given by Equation (2). In this study, *R* is chosen as 50 Ω, resulting in V_diff_ = 1 V after the current-to-voltage conversion. With R_1_ = R_2_ = 500 Ω, the gain of the differential amplifier is 1, and the output single-ended voltage signal has a peak amplitude of 2 V (4 Vpp).

(1) $$V_{diff}=I_{diff}R$$

(2) $$V_{O}=\frac{2V_{diff}R_{2}}{R_{1}}$$

In this study, the ERT hardware framework was redesigned at the main-control, excitation, sampling, and electrode-selection levels. This modular description allows the roles of the main functional blocks to be discussed under the same sensor configuration. The available experiments comprise a cross-prototype signal-quality comparison emphasizing the excitation path and an end-to-end system comparison. They do not constitute a filter-only switching test or a complete factorial ablation. Because the second-order and seventh-order filters are integrated into separate fabricated PCBs, replacing only the filter while holding the remaining hardware unchanged would require board redesign, refabrication, and revalidation.

The DAC output and high-speed digital switching can introduce harmonic and out-of-band noise into the excitation path. To improve excitation purity, this study uses a high-order passive low-pass filter. Candidate filter responses are compared in Table 2 according to passband flatness, transition-band steepness, and ripple characteristics.

In practical ERT measurement systems, signals measured at remote electrodes are attenuated and easily affected by interference. A flat passband is therefore important for maintaining a stable excitation amplitude, while strong out-of-band suppression is needed to reduce high-frequency components that can be coupled into the sampling path. For this reason, a Butterworth response was selected for the excitation low-pass filter.

Traditional conductivity or impedance tomography prototypes commonly employ second-order Sallen-Key (SK) active low-pass filters because they are simple and inexpensive. However, their limited order results in a gradual roll-off and insufficient suppression of high-frequency distortion. This study therefore uses a seventh-order LC low-pass filter based on the Butterworth prototype design. The higher order provides a steeper transition band while preserving a flat passband, thereby improving waveform fidelity without adding active-device bandwidth limitations to the excitation path.

**Figure 6:**
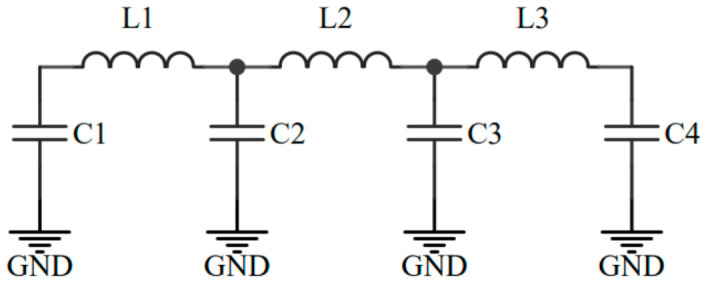
Seventh-order constant-k Butterworth prototype filter.

The seventh-order LC low-pass filter is derived from the normalized Butterworth prototype shown in Figure 6. Let gk denote a normalized prototype coefficient. For the shunt-capacitor/series-inductor ladder, frequency and impedance scaling to the cutoff frequency fc and characteristic impedance Z_0_ are performed using Equation (3).

(3) $$C_{k}=\frac{g_{k}}{2\pi f_{c}Z_{0}},\;L_{k}=\frac{Z_{0}g_{k}}{2\pi f_{c}}$$

Substituting fc = 10 MHz and Z_0_ = 50 Ω gives the calculated component values listed in Table 3; the nearest available components were then selected for implementation.

(4) $$FEXT=\frac{l}{2vt_{r}}\left(\frac{C_{m}}{C_{s}}-\frac{L_{m}}{L_{s}}\right),$$

(5) $$NEXT=\frac{1}{4}\left(\frac{C_{m}}{C_{s}}+\frac{L_{m}}{L_{s}}\right),$$

(6) $$v=c/\sqrt{\varepsilon_{r}},$$

In Equations (4)–(6), FEXT and NEXT are the dimensionless far-end and near-end crosstalk coupling coefficients, respectively; Cm and Lm are the mutual capacitance and mutual inductance per unit length between adjacent traces; Cs and Ls are the trace-to-reference capacitance and trace self-inductance per unit length; l is the coupled trace length (m); tr is the incident-signal rise time (s); v is the propagation velocity (m/s); c is the speed of light (m/s); and εr is the relative permittivity of the PCB dielectric (dimensionless). When needed, the coupling coefficients can be expressed as crosstalk levels using 20 log10|FEXT| or 20 log10|NEXT| in decibels.

The implemented cutoff frequency is 10 MHz and the termination impedance is 50 Ω. The nearest available components in Table 3 differ from the prototype-scaled values by no more than 5.9%. Because the network is passive, it has no closed-loop stability issue; potential resonance and peaking are limited by the 50 Ω source and load terminations. A formal Monte Carlo tolerance analysis and direct network-analyzer uncertainty assessment were not performed, so the simulated attenuation values are nominal predictions rather than uncertainty bounds.

**Figure 7:**
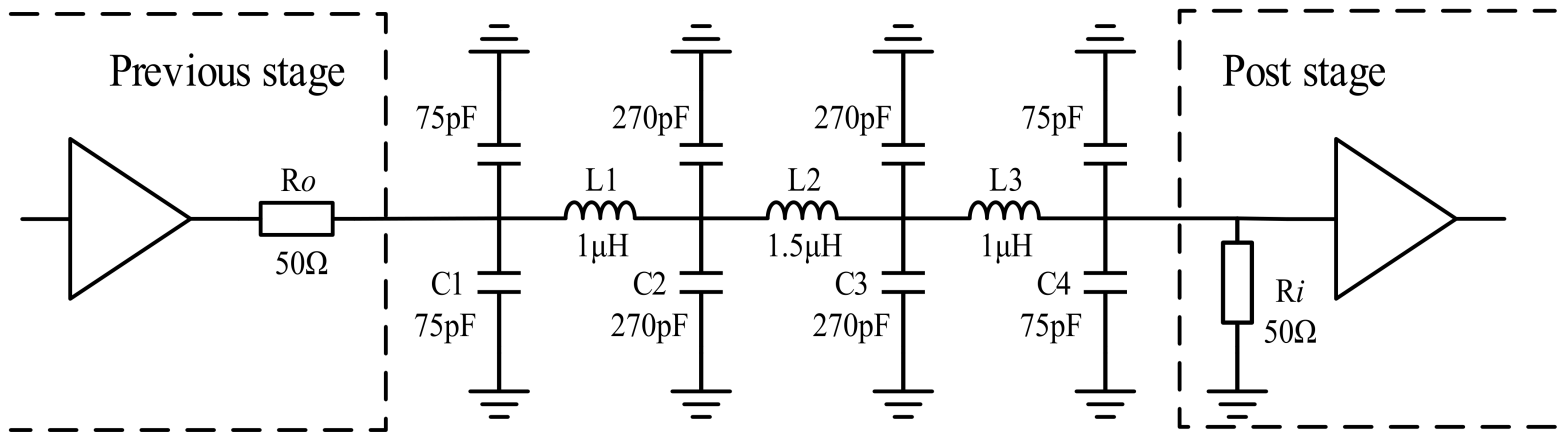
Impedance-matching design for the filter.

To mitigate the impact of parasitic inductance on PCB routing [19], this study employs two capacitors in parallel, each with a capacitance value half of the original, as depicted in Figure 6. Because components with the exact calculated capacitance and inductance values are difficult to obtain, a practical approximation is used in this study, and the implemented values are listed in the Implemented value column of Table 3. Given the characteristic impedance of 50 Ω for the filter, impedance matching is required at the input and output stages of the filter, as illustrated by Ro and Ri in Figure 7.

**Figure 8:**
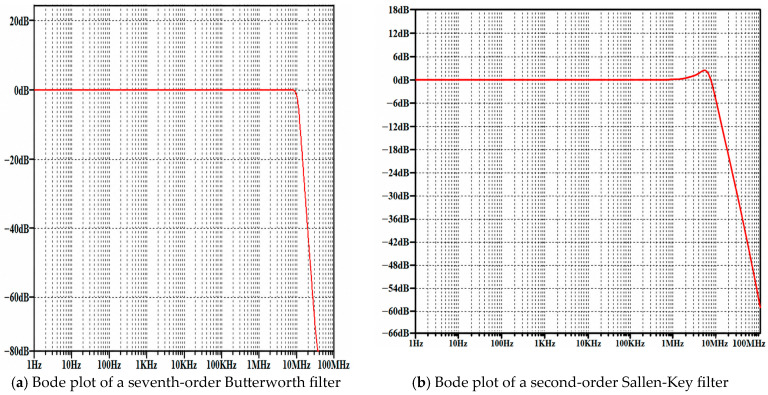
Simulated magnitude responses of the low-pass filters (frequency in Hz; magnitude in dB).

The nominal frequency responses of the baseline second-order Sallen-Key active low-pass filter and the proposed seventh-order Butterworth LC filter were first compared in LTSpice. The Bode plots are shown in Figure 8 and the nominal parameters are compared in Table 4. Section 3.3 separately reports waveforms, calculated SNR, and total harmonic distortion measured from the two fabricated prototypes. These hardware measurements compare the complete excitation paths; they should not be interpreted as a same-board filter-switching experiment, and the simulation and hardware results are not treated as interchangeable.

The nominal simulation predicts approximately 20.4 dB additional attenuation at 2f_c_ (20 MHz) and approximately 81.1 dB additional attenuation at 10f_c_ (100 MHz) for the seventh-order Butterworth filter relative to the second-order Sallen-Key filter. These values describe the simulated filters and support the choice of topology; the measured signal-quality change is evaluated independently in Section 3.3.

The Butterworth filter also avoids the high-frequency gain degradation associated with operational-amplifier bandwidth limitations in active SK filters. Although a passive LC implementation requires impedance matching and careful component selection, it provides greater high-frequency stability for the excitation chain in this prototype.

In summary, the seventh-order Butterworth filter is a component-level modification intended to improve excitation waveform fidelity and suppress out-of-band harmonics. The nominal simulation supports the topology choice, while the hardware measurements describe the signal quality of the two assembled excitation paths. Because the filters are fixed on separate PCBs, the present experiment cannot isolate the filter contribution from all associated analog implementation differences.

**Figure 9:**
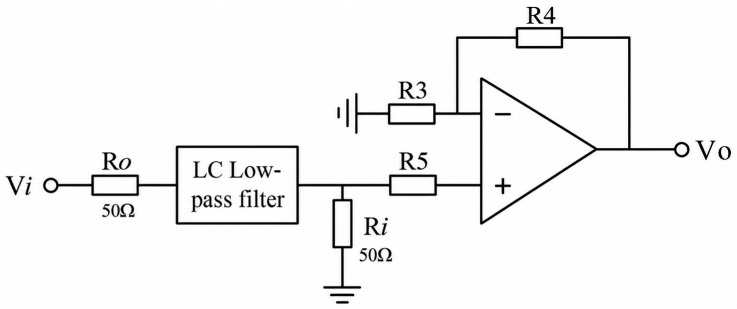
Output stage of the excitation module.

The filtered excitation signal is amplified by the non-inverting output stage shown in Figure 9, and its output is described by Equation (7). With R_3_ = R_4_ = 500 Ω, the stage gain is 2; a 2 V peak sinusoid therefore corresponds to 4 Vpp, consistent with the waveform-test condition stated above.

(7) $$V_{O}=V_{i}(1+R_{4}/R_{3})/2$$

### 2.3. Sampling Circuit

The primary function of the sampling section is to measure the current on the test electrode through current-sensing resistors, condition and digitize the resulting voltage, and transfer the samples to the main control module. According to the Nyquist sampling theorem [21], the sampling frequency should satisfy Equation (8).

(8) $$f_{S}\geq 2f_{\max}$$

In Equation (8), fs is the sampling frequency in samples per second (S/s, numerically expressed in Hz), and fmax is the highest frequency component of interest in hertz (Hz). For the 100 kHz excitation case, the Nyquist lower bound is 200 kS/s.

**Figure 10:**
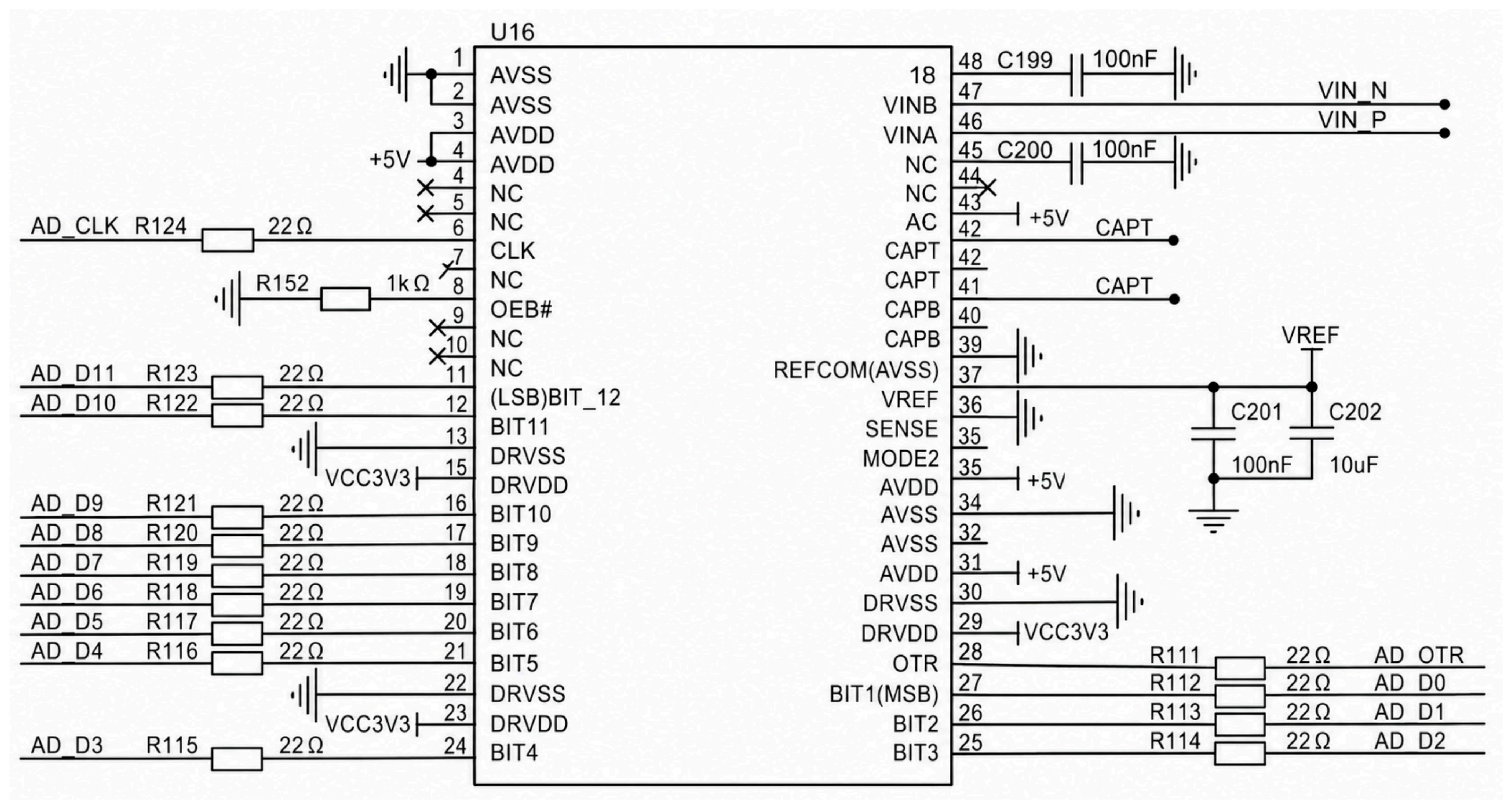
AD9226 A/D converter and peripheral circuit.

Therefore, the sampling rate should exceed 200 kSPS. In practice, a sampling rate of at least ten times the excitation frequency is preferred to support reliable amplitude and phase extraction. Although the ZYNQ-7000 APS-SoC integrates a 12-bit XADC, its 1 MSPS sampling rate is insufficient for the high-rate sampling strategy used in this study. Therefore, an external AD9226 high-speed ADC is used for signal acquisition, as shown in Figure 10.

The AD9226 provides 12-bit conversion at a maximum sampling rate of 65 MSPS. In the prototype tests, the ADC clock was set to 20 MSPS, which is more than sufficient for 100–160 kHz excitation and remains safely below the device limit. The statement regarding PLL multiplication in the PL therefore describes internal logic timing and does not override the AD9226 sampling-rate specification.

### 2.4. Filter-Amplifier Circuit

**Figure 11:**
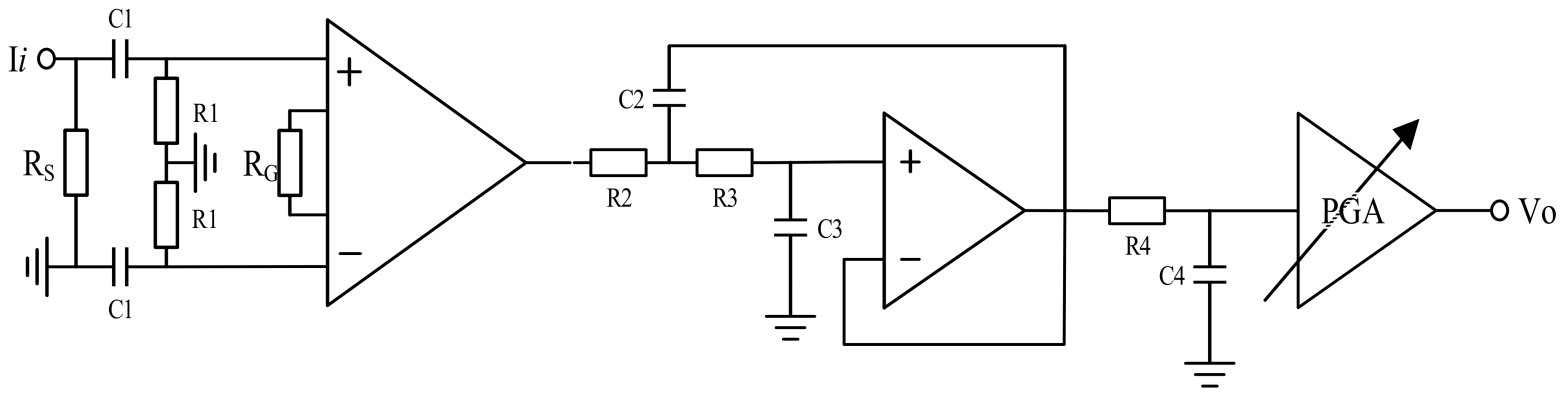
Analog front-end filtering and variable-gain amplification circuit.

The signals measured at the electrodes are typically weak, particularly at electrodes opposite the excitation side, with amplitudes of only a few hundred microvolts. Consequently, these signals can be buried in noise and therefore require filtering and amplification through the signal-conditioning circuitry shown in Figure 11.

**Figure 12:**
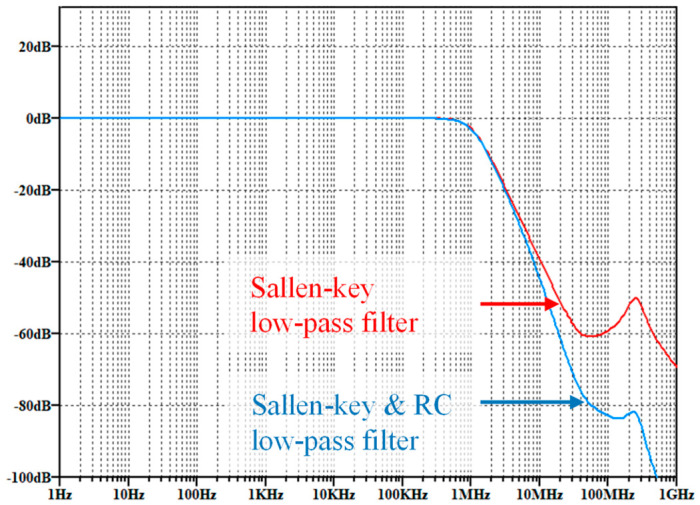
Simulated magnitude responses of the Sallen-Key filter and Sallen-Key-plus-RC filter.

RS is the current-sensing resistor and is selected to be much smaller than the input impedance of the following stage. The AD8249 instrumentation amplifier provides an initial gain of 5 V/V. The input high-pass and downstream RC stages were designed using fc = 1/(2πRC), and the active low-pass stage follows the standard Sallen-Key relations (see Figure 12). The high-pass corner was placed well below the 100–160 kHz excitation band while attenuating 50/60 Hz interference. The cascaded Sallen-Key and RC low-pass stages were selected to produce the simulated 30.195–956.280 kHz passband and suppress high-frequency feedthrough. The THS7001 programmable-gain range (−22 to 20 dB) was then selected so that weak channels exceed the ADC quantization level while strong channels remain within the 2 Vpp AD9226 input range. Thus, the resistance, capacitance, gain, and corner-frequency choices are linked to the excitation band, the expected electrode-signal range, and the ADC dynamic range.

**Figure 13:**
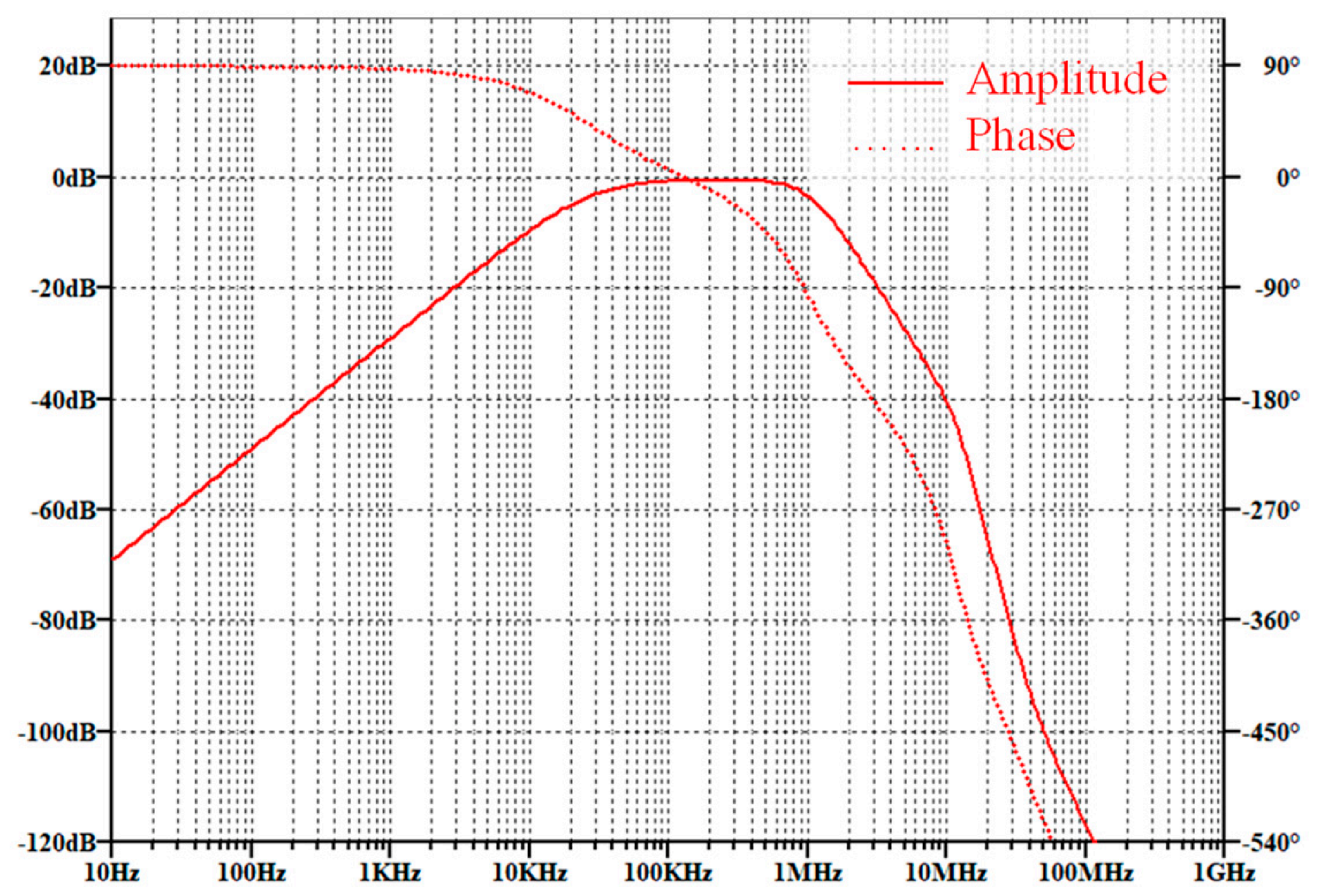
Simulated magnitude and phase responses of the analog front end.

When the input range of the AD9226 is set to 2 Vpp, its voltage resolution is in microvolts. Thus, the analog front end requires an appropriate amplification factor to ensure that the input signal exceeds the voltage resolution of the analog-to-digital converter. The parameters of the sampling front-end circuitry chosen in this study are selected according to the AD9226 input range and simulated in LTSpice. The magnitude and phase frequency responses of the filters are shown in Figure 13.

Figure 13 shows the magnitude and phase frequency responses of the analog front-end. The passband frequency of the filter ranges from 30.195 to 956.280 kHz, with a phase shift of 6.026° at 100 kHz. The instrumentation amplifier is set to a constant gain of 5 V/V, while the subsequent stage implemented with the THS7001 variable-gain amplifier provides gains ranging from −22 dB to 20 dB. Consequently, the analog front end can provide a maximum gain of 50 V/V. This configuration amplifies the signal from the most distant electrode to several hundred millivolts while attenuating signals from nearby electrodes to prevent saturation.

## 3. Experimental Evaluation

### 3.1. Experimental Setup and Timing Sequence

**Figure 14:**
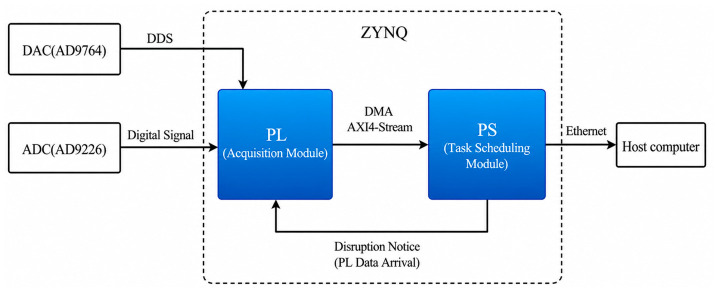
Experimental setup and qualitative acquisition/processing sequence.

The functional sequence for each switching state is PS parameter update; PL switch-word update; analog settling; 2048-sample ADC acquisition; FIFO buffering and DMA transfer to DDR; PS packetization; Ethernet transmission; and host-side FFT amplitude extraction and reconstruction. Figure 14 depicts this qualitative sequence. Only end-to-end latency was measured, from the acquisition trigger to the availability of one reconstructed frame on the host computer. The available laboratory instruments did not provide a common hardware trigger or synchronized timestamping across the legacy FPGA/RS-485 controller, the ZYNQ PL/PS, the Ethernet interface, and the host computer. Component-specific timestamps were therefore not recorded, and the reported 2.1 ms value cannot be decomposed or assigned numerically to any single stage.

### 3.2. Electrode Switching Mode and Acquisition Sequence

The system uses an adjacent electrode-switching strategy for the 16-electrode ERT sensor. In one frame, the excitation pair is sequentially switched as (E_1_,E_2_), (E_2_,E_3_), …, (E_16_,E_1_). For each excitation pair, the voltage responses of the remaining adjacent electrode pairs are acquired after a short settling interval; drive-pair and reciprocal measurements are excluded. Thus, 13 valid voltage measurements are retained for each excitation state, and one complete frame contains 16 × 13 = 208 independent boundary measurements. The PL updates the switching control word, waits for analog settling, triggers ADC acquisition, and stores the data in FIFO before DMA transmission.

### 3.3. Excitation Waveform, Frame Rate, and Signal-Quality Tests

**Figure 15:**
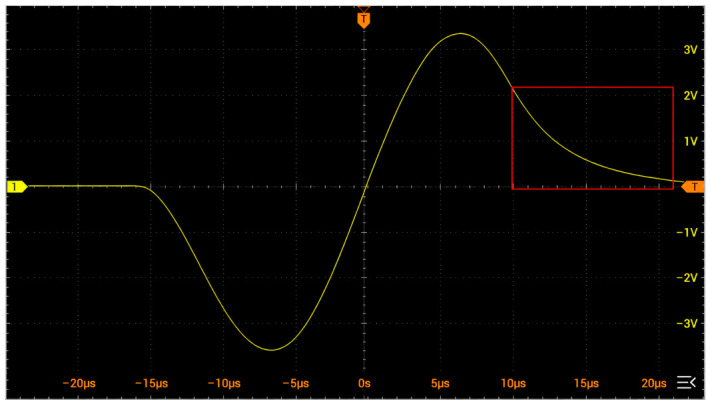
Excitation waveform of the proposed APS-SoC-based ERT system.

**Figure 16:**
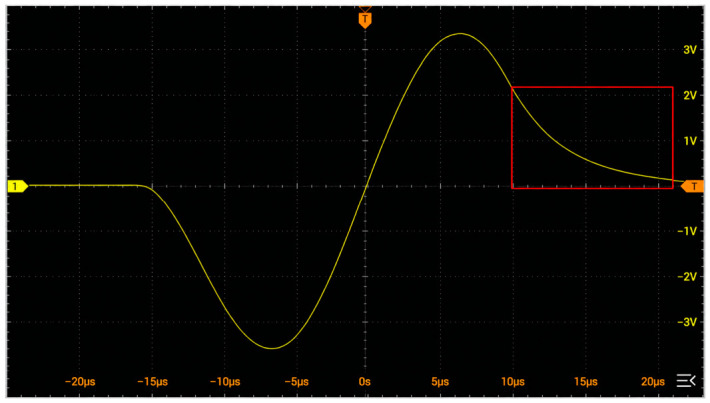
Excitation waveform of the baseline FPGA-based ERT system.

For the waveform comparison, the excitation frequency was set to 100 kHz and the main control board output was measured using a Poynting DHO1074 oscilloscope with 12-bit vertical resolution. Figure 15 and Figure 16 show the proposed and baseline excitation waveforms, respectively.

The baseline waveform shows visible end-of-cycle distortion in the marked region of Figure 16. In contrast, the proposed path produces a waveform closer to a sinusoid under this test condition, consistent with the higher-order excitation filter and impedance-matched output stage.

Both systems were tested in ten repeated trials on the same 16-electrode measurement field. Each round contained 100 frames; the time values in Table 5 therefore represent the acquisition and delivery time for one 100-frame cycle. Frame rate was calculated as 100 divided by the corresponding cycle time. Across the ten rounds, the baseline achieved 58.23 ± 1.88 FPS and the proposed system achieved 123.02 ± 1.62 FPS (mean ± sample standard deviation).

To describe the excitation-path signal-quality difference separately from the frame-rate comparison, one representative static hardware record was acquired at 160 kHz with the AD9764 and AD9226 clocks set to 20 MHz. The baseline RS-485 link was configured at 2 Mbps for the end-to-end comparison. The waveform, calculated SNR, and total harmonic distortion values in Table 6 were obtained from the two fabricated prototypes and are not LTSpice outputs. The second-order and seventh-order filters are permanently integrated into their respective PCB excitation paths; a controlled same-board filter switch would require redesign and refabrication. Accordingly, this is a cross-prototype excitation-path comparison rather than a filter-only ablation. The amplitude-stability SNR metric was calculated using Equation (9).

(9) $$SNR=20\log_{10}(\frac{\bar{x}}{\sigma_{x}})$$

In Equation (9), $\bar{x}$ is the mean of the extracted steady-state signal-amplitude samples and σx is their standard deviation. This ratio describes amplitude stability in the selected record and should not be interpreted as a broadband, instrument-limited spectral SNR specification. Repeated raw spectra, a filter-switching dataset, and a formal uncertainty budget were not available for the present revision. Therefore, the reported values are retained only as descriptive single-condition observations and are not used to establish a general performance specification.

Under the stated single-condition test, the calculated amplitude-stability SNR values were 65 dB for the baseline path and 100 dB for the proposed path, while total harmonic distortion decreased from 15.2% to 7.5%. The 7.5% value is not presented as a universal acceptance threshold for ERT; it indicates a relative difference between the two assembled laboratory prototypes and was sufficient for stable amplitude extraction in this experiment. Because a same-board filter-only comparison and repeated matched spectra were unavailable, these values cannot be used to quantify the isolated contribution of the seventh-order filter. Metrological interpretation would require repeated spectra, bandwidth-defined noise analysis, component-tolerance characterization, and an uncertainty budget.

Table 5 shows that the proposed system achieved an average frame rate of 123.02 FPS compared with 58.23 FPS for the baseline. The frame-rate gain is associated with the combined PL/PS data path, DMA buffering, and Gigabit Ethernet interface. The legacy and proposed communication paths reside on different controller boards, use different firmware and buffering mechanisms, and could not be driven by the same data source under a common synchronized trigger with the available laboratory equipment. An isolated RS-485-versus-Gigabit-Ethernet timing test was therefore not performed. Consequently, the present data do not quantify the independent contribution of the ZYNQ architecture, DMA organization, or communication-interface replacement. The Butterworth filter is not expected to increase data-transfer speed and is discussed only in relation to excitation-path signal quality.

### 3.4. Image Reconstruction Method and Quantitative Metrics

For image reconstruction, the linearized ERT model is written as

(10) ΔV=JΔσ+n

where Δ*V* is the boundary-voltage variation, *J* is the sensitivity matrix, Δ*σ* is the conductivity variation, and *n* denotes measurement noise. The iterative Tikhonov update is obtained by minimizing

(11) $${\left.\left\|J\Delta \sigma -\Delta V\right.\right\|}_{2}^{2}+\lambda^{2}{\left.\left\|L\Delta \sigma \right.\right\|}_{2}^{2}$$

which gives

(12) $$\Delta \sigma ={\left(J^{T}J+\lambda^{2}L^{T}L\right)}^{-1}J^{T}\Delta V$$

In this study, *L* is taken as an identity or smoothness matrix according to the reconstruction mesh, and the regularization parameter *λ* is selected using the L-curve rule and then kept unchanged for the comparison between the baseline and proposed systems.

The host-side reconstruction was implemented by the authors using in-house MATLAB (R2025b) scripts rather than a third-party ERT reconstruction package. The scripts perform FFT-based amplitude extraction, assemble the geometry-specific sensitivity matrix, select λ by the L-curve rule, and solve the regularized update in Equation (12). The same code, mesh, preprocessing, and regularization settings were used for the baseline and proposed data sets.

Although Equation (12) is the conventional Tikhonov inverse, the forward/sensitivity model was constructed for the actual measurement geometry. Specifically, J was calculated on a two-dimensional circular finite-element mesh matching the basin cross-section, the 16 ring-arranged boundary electrodes, and the adjacent drive/measurement protocol. Thus, the inversion did not use a flat or planar sensitivity model; only the regularized algebraic solver is conventional. No geometry-specific modification of the Tikhonov objective was required because the geometry enters through J and the mesh. The reconstruction remains a two-dimensional cross-sectional approximation, and electrode contact impedance and out-of-plane effects were not explicitly modeled.

**Figure 17:**
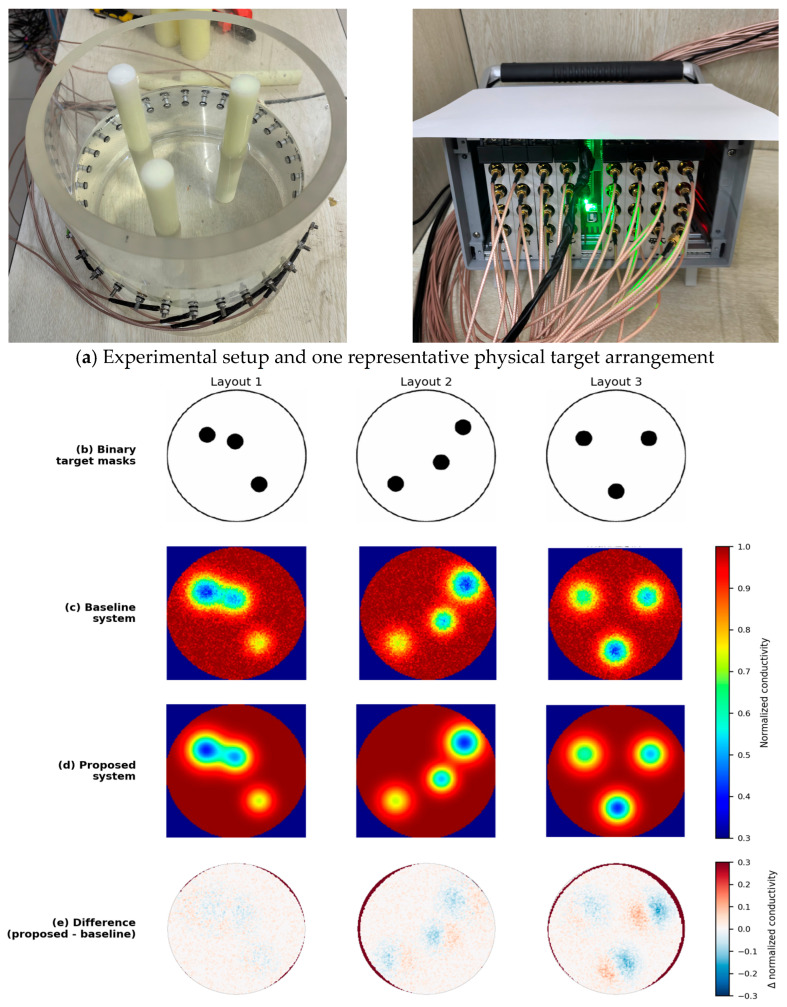
Experimental setup and direct image comparison: (**a**) experimental setup and one representative target arrangement; (**b**) binary target masks; (**c**) baseline reconstructions; (**d**) proposed-system reconstructions; and (**e**) pixel-wise difference maps, Δσ_norm = σ_norm, proposed − σ_norm, baseline. Panels (**c**,**d**) use identical display settings and a shared normalized-conductivity scale of 0.3–1.0 (dimensionless); panel (**e**) uses a symmetric scale of −0.3 to 0.3 (dimensionless).

Imaging performance was evaluated in a circular water basin with 16 ring-arranged electrodes using three geometric arrangements of the same three insulated cylindrical rods, as shown in Figure 17. Both systems used the same host-side Tikhonov reconstruction method, mesh, L-curve-selected regularization parameter, excitation condition, and preprocessing. Rod positions and diameters were measured to generate geometric binary target masks. The medium was municipal tap water (approximately 192 μS/cm), the excitation frequency was 160 kHz, the sampling rate was 20 MS/s, and each boundary measurement contained 2048 samples. Although three layouts are displayed, the validation remains limited to one medium, one inclusion material and one inclusion shape, one three-inclusion complexity level, and one representative excitation frequency. For direct visual comparison, the baseline and proposed reconstructions are displayed in paired rows with identical interpolation, spatial limits, color map, and a shared normalized-conductivity range of 0.3–1.0.

The metrics in Table 7 were computed from normalized reconstructed images. SFDR was calculated from the sampled waveform spectrum as the difference between the fundamental and largest spurious component. SSIM and RMSE were computed against the measured-geometry binary masks, which are surrogate target maps rather than direct measurements of the continuous conductivity field. Edge sharpness was calculated as the normalized mean gradient magnitude along the target boundaries. End-to-end latency was measured from acquisition triggering to the availability of one reconstructed frame on the host computer. No image post-processing was applied. The manuscript reports one descriptive value per metric for the displayed data set; per-layout confidence intervals and independent repeated-acquisition variance were not available. The color map represents normalized conductivity (dimensionless); lower values correspond to the insulated, low-conductivity rods relative to the water background. Because the images are normalized, the color bar does not represent absolute conductivity in S/m. Figure 17e shows the pixel-wise difference Δσ_norm = σ_norm, proposed − σ_norm, baseline using a symmetric range of −0.3 to 0.3.

As shown in Figure 17b–d, the reconstructed low-conductivity regions are spatially consistent with the measured rod positions across the three displayed arrangements. The same reconstruction algorithm and regularization parameter were used for both systems. Consequently, differences in SSIM, RMSE, and edge sharpness should be interpreted primarily as effects of cleaner boundary-voltage measurements, not as an improvement in the reconstruction algorithm. Figure 17e provides the pixel-wise proposed-minus-baseline difference for each layout; the localized positive and negative bands around inclusion boundaries make the reduced blurring and altered transition profiles more visible. Because the plotted quantity is normalized conductivity, these difference maps are dimensionless and are intended as a direct visual comparison rather than an absolute conductivity-error map. Lower communication latency reduces real-time delivery delay but does not by itself improve image similarity.

The baseline reconstructions show greater boundary blurring and peripheral artifacts, whereas the proposed measurement chain yields sharper target transitions in the displayed tests. These observations should not be generalized beyond the three rod arrangements without experiments involving other conductivity contrasts, inclusion types, noise levels, and operating frequencies, together with residual maps, sensitivity analysis, and repeated image statistics.

A higher SFDR indicates cleaner sampled signals. The increase in SSIM from 0.72 to 0.94 and the decrease in RMSE from 0.184 to 0.072 indicate closer agreement with the geometric binary masks for the displayed data set. Because per-layout values and repeated-acquisition variance are unavailable, these metrics should be interpreted descriptively rather than as statistically generalizable performance estimates.

Overall, the results support a specific conclusion: for the tested laboratory configurations, the optimized measurement chain provides cleaner boundary-voltage data and faster host delivery under unchanged reconstruction settings.

Table 8 shows modest PL utilization, leaving implementation headroom for future embedded demodulation or reconstruction acceleration. The estimated payload rate is approximately 104.8 MB/s, or 26.2% of a 32-bit, 100 MHz AXI4-Stream path, while remaining within the practical range of Gigabit Ethernet after protocol overhead. PS CPU load and board-level power were not measured. The available laboratory instruments also did not support synchronized, stage-resolved power and timing measurements across the two different controller boards. The integrated ZYNQ device may reduce inter-chip transfers, whereas the Gigabit Ethernet PHY and revised analog circuitry also consume power. Accordingly, neither the direction nor the magnitude of the net power change can be established and no component-level latency or energy-efficiency claim is made.

## 4. Conclusions and Outlook

This study implemented a system-level optimization of a 16-electrode ERT measurement platform using ZYNQ-7020 PL/PS cooperation, AXI/DMA data movement, Gigabit Ethernet, and a seventh-order Butterworth excitation filter. In the controlled laboratory comparison, frame rate increased from 58.23 ± 1.88 FPS to 123.02 ± 1.62 FPS, and end-to-end latency decreased from 5.2 ms to 2.1 ms. A representative excitation-path test also showed higher calculated amplitude stability and lower total harmonic distortion.

With identical host-side Tikhonov reconstruction settings, the proposed measurement chain produced higher SFDR and improved descriptive image metrics across the displayed three-rod layouts. These differences are attributed to cleaner boundary-voltage data rather than a new reconstruction method. Because the controller/data path, communication interface, and analog filtering changed together, the present study does not isolate every contribution or establish universal state-of-the-art superiority.

The remaining limitations include restricted phantom diversity; the absence of component-wise latency, synchronized link-only timing, PS-load, and power measurements; the lack of repeated spectral and image uncertainty analysis; and the absence of a full factorial ablation. In particular, the excitation filters are fixed on separate fabricated PCBs, and the legacy and proposed communication paths cannot be measured under a common synchronized trigger with the current laboratory setup. Future work will use replaceable filter modules and synchronized instrumentation to separate analog, controller, DMA, and communication contributions. Future work will also evaluate additional conductivity distributions, inclusion types, noise levels, and excitation frequencies; report per-layout repeated statistics, raw spectra, residual and sensitivity maps; measure PS load and board-level power; and implement ARM/PL-accelerated demodulation or reconstruction toward a standalone embedded sensor.

## Figures and Tables

**Table 1:** Comparison with representative FPGA- or APS-based ERT/EIT systems.

Study	Platform	Electrodes	Excitation	Filter/Front End	Main Reported Metrics
Darnajou et al. [23]	FPGA-based EIT	16 planar electrodes	Multifrequency, simultaneous (FDM/ONE-SHOT)	N/R	SNR 55.6–69.1 dB; frame rate 3906 fps
Xu et al. [24]	FPGA-based multifrequency EIT	16 electrodes	Multifrequency with reference signal measurement	Butterworth low-pass filters	Amplitude and phase calibrated; wideband SNR 71.55 (amplitude), 64.12 dB (phase)
Vejar and Rymarczyk [25]	FPGA-based electrical tomography	N/R	Single—or configurable waveforms; reconfigurable in real-time	N/R	Standard EIT reconstruction; potential profile/impedance metrics
Rifai et al. [26]	FPGA-psEIT	Planar electrodes	Single-frequency constant current; DFHC pump	Programmable front-end measurement (PFM)	SNR 89 dB; Na^+^ skin imaging; voltage measurement accuracy
This work	ZYNQ-7020 APS-SoC-based ERT	16 electrodes	Single—or multi-phase sinusoidal signals; fully programmable	7th-order Butterworth filter	123.02 ± 1.62 FPS (n = 10); representative calculated amplitude-stability SNR = 100 dB at 160 kHz; SFDR 95 dB; SSIM 0.94; latency 2.1 ms

**Table 2:** Filter types and characteristics.

Type	Characteristic
Butterworth	The passband is flat, but the transition band is broad
Chebyshev	The transition band is narrow, but there are ripples in the passband or stopband
Bessel	The passband is flat and the group delay is constant, but the transition band is broad
Elliptic function	For a given performance specification, the required order is low, but ripples occur in the passband and stopband

**Table 3:** Calculated and implemented component values of the seventh-order Butterworth filter.

Component	Calculated Value	Implemented Value
C1	141.648 pF	2 × 75 pF
C2	573.594 pF	2 × 270 pF
C3	573.594 pF	2 × 270 pF
C4	141.648 pF	2 × 75 pF
L1	992.331 nH	1 μH
L2	1.592 μH	1.5 μH
L3	992.331 nH	1 μH

**Table 4:** Simulated performance parameters of the two filter topologies.

Metric	2nd-Order Sallen-Key (Q = 1.2)	7th-Order Butterworth
Passband ripple (0 − f_c_)	2.941 dB	0.412 dB
Magnitude at 2 × f_c_ (20 MHz)	−21.710 dB	−42.144 dB
Magnitude at 10 × f_c_ (100 MHz)	−58.943 dB	−140.030 dB

**Table 5:** Repeated 100-frame timing and frame-rate results.

Baseline FPGA-Based ERT System			Proposed APS-SoC-Based ERT System	
No.	Time for 100 Frames (s)	Frame Rate (FPS)	Time for 100 Frames (s)	Frame Rate (FPS)
1	1.87	53.48	0.80	123.46
2	1.68	59.52	0.80	123.46
3	1.69	59.17	0.79	126.58
4	1.68	59.52	0.80	123.46
5	1.70	58.82	0.80	123.46
6	1.76	56.82	0.83	120.48
7	1.69	59.17	0.82	121.95
8	1.73	57.80	0.80	123.46
9	1.71	58.48	0.82	121.95
10	1.68	59.52	0.82	121.95
-	1.72 ± 0.06	58.23 ± 1.88	0.81 ± 0.01	123.02 ± 1.62

**Table 6:** Representative signal-quality measurements.

Index	Baseline Path	Proposed Path
Calculated amplitude-stability SNR (dB)	65	100
Total harmonic distortion (%)	15.2	7.5

**Table 7:** Descriptive metrics for the displayed three-layout, three-rod imaging data set.

Metric	Baseline FPGA-Based ERT System	Proposed APS-SoC-Based ERT System	Change
SFDR (dB)	68	95	+27
Edge sharpness (%)	53	77	+24
SSIM	0.72	0.94	+30.6%
RMSE	0.184	0.072	−60.9%
Latency (ms)	5.2	2.1	−59.6%

**Table 8:** ZYNQ-7020 implementation metrics and data-path analysis.

Metric	Value/Calculation	Remarks
Device	ZYNQ-7020 APS	PL for acquisition/control; PS for configuration and communication
PL clock	100 MHz system clock; PLL available for internal timing	ADC sampling is limited by AD9226 to ≤65 MS/s
Experimental ADC sampling	20 MS/s	Used for the 160 kHz imaging experiment
LUT utilization	7483 LUTs/53,200 LUTs = 14.07%	Obtained from Vivado implementation report
BRAM usage	12 blocks/140 blocks = 8.57%	Obtained from Vivado implementation report
DSP slices	2 slices/220 slices = 0.91%	Obtained from Vivado implementation report
BUFG usage	2/32 = 6.25%	Obtained from Vivado implementation report
IO usage	28 pins/125 pins = 22.40%	Obtained from Vivado implementation report
AXI4-Stream theoretical capacity	32 bits × 100 MHz = 400 MB/s	Used to estimate internal data-path utilization
Estimated AXI utilization	104.8 MB/s/400 MB/s = 26.2%	Calculated from present frame size and frame rate
Raw data per frame	16 × 13 × 2048 × 2 bytes = 0.852 MB	Adjacent mode, 2048 samples, 16-bit storage
Average payload rate	0.852 MB × 123.02 FPS = 104.8 MB/s	Below the theoretical 125 MB/s Gigabit Ethernet limit; packet overhead should be managed
Measured latency	2.1 ms for the improved system; 5.2 ms for the existing system	End-to-end latency from Table 7

## Data Availability

The original contributions presented in this study are included in the article. Further inquiries can be directed to the corresponding author.
